# The concentration of D-dimers in portal blood positively correlates with overall survival in patients with non-resectable pancreatic cancer

**DOI:** 10.1186/s12957-017-1291-4

**Published:** 2017-12-16

**Authors:** Adam Durczynski, Aleksander Skulimowski, Piotr Hogendorf, Dariusz Szymanski, Anna Kumor, Konrad Marski, Siri Øvereng Juliebø, Grazyna Poznanska, Janusz Strzelczyk

**Affiliations:** 1 Department of General and Transplant Surgery, Barlicki Teaching Hospital, Medical University of Lodz, Kopcinskiego Street 22, 90-153 Lodz, Poland; 2 Department of Pulmonology and Allergy, Barlicki Teaching Hospital, Medical University of Lodz, Lodz, Poland; 3 Department of Anaesthesiology and Intensive Care, Barlicki Teaching Hospital, Lodz, Poland

**Keywords:** Pancreatic cancer, D-dimers, Portal blood, Overall survival

## Abstract

**Background:**

Several recent studies provide evidence that D-dimer (DD) concentration in peripheral blood correlates negatively with overall survival (OS) in patients with pancreatic ductal adenocarcinoma (PDAC). Contrarily, there are recent evidence indicating that preoperative plasma fibrinogen, but not D-dimer might represent a prognostic factor in non-metastatic gastrointestinal cancers.

**Methods:**

In a single-center prospective study, we enrolled 62 patients undergoing surgery for pathologically confirmed PDAC without detectable venous thrombosis. Intraoperatively, the sample of the blood from the portal vein was obtained. DD concentration in these samples was measured. Patients were followed postoperatively until time of death from any cause.

**Results:**

We found that OS for patients with portal blood DD values above 2700 (ng/mL) (*n* = 22 from 62 patients) was higher by 158% than that for the patients (*n* = 42) with DD values ≤ 2700 (416 days versus 161 days, *p* = 0.05). On the contrary to the studies investigating DD concentration in peripheral blood, we have found that patients with higher DD level in the portal vein had longer mean OS than patients with lower ones.

**Conclusions:**

Further investigation is necessary both to confirm our results in a larger patient population and to elucidate the mechanism for the correlation between portal blood D-dimer concentrations and survival time. Along with other authors, we conclude that portal circulation is characterized by unique, biological environment that requires further evaluation.

## To the Editor:

Surgery remains the only potentially curative treatment for pancreatic ductal adenocarcinoma (PDAC); however, postoperative prognosis is variable and difficult to estimate. CA 19-9 has demonstrated clinical utility in this regard [1] but suffers from several limitations such as unclear cut-off points and inapplicability to Lewis antigen non-secretors. Therefore, there is a need to validate other prognostic biomarkers. Several recent studies provide evidence that D-dimer (DD) concentration in peripheral blood correlates negatively with overall survival (OS) in patients with PDAC [2, 3]. Contrarily, there is recent evidence indicating that preoperative plasma fibrinogen, but not D-dimer might represent a prognostic factor in non-metastatic gastrointestinal cancers [4]. In our previous research, we have extended investigations to DD concentration in bile, urine, and portal vein blood of patients with pancreatic cancer [5]. Following statistical analysis of our results, we have decided to investigate correlation between DD concentration in portal vein and overall survival (OS).

In a single-center prospective study, we enrolled 62 patients undergoing surgery for pathologically confirmed PDAC without detectable venous thrombosis. In all of them, during the surgery, tumor was found to be locally advanced and non resectable, but no metastatic lesions were identified. Such group of patients is characterized by lower predicted OS and extremely poor prognosis [3].

Intraoperatively, the sample of the blood from the portal vein was obtained. DD concentration in these samples was measured using VIDAS D-Dimer Exclusion II assay (bioMerieux, France). All of them were submitted to postoperative chemotherapy with gemcitabine. Patients were followed postoperatively until time of death from any cause. The study was approved by Ethics Committee of Medical University of Lodz in Poland. All statistical calculations were performed using SPSS version 20.0 for Windows (IBM Corporation, Armonk, NY).

**Fig. 1:**
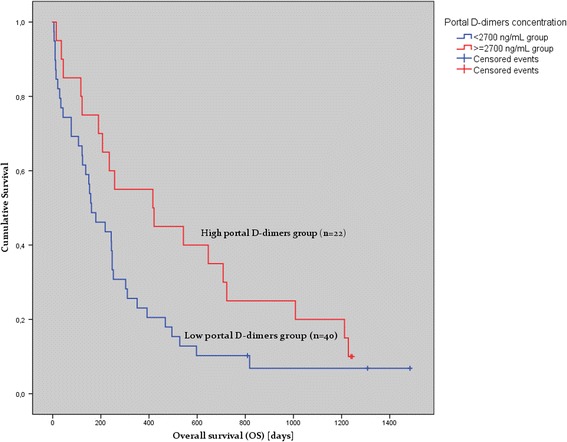
Correlation of portal blood D-dimers with overall survival of patients with nonresectable pancreatic cancer. Kaplan-Meier curve

We found that OS for patients with portal blood DD values above 2700 (ng/mL) (*n* = 22 from 62 patients) was higher by 158% than that for the patients (*n* = 42) with DD values ≤ 2700 (416 versus 161 days, *p* = 0.05) (Fig. 1). No statistically significant interaction was observed between the bile, urine and peripheral blood DD levels and OS of these patients.

On the contrary to the studies investigating DD concentration in peripheral blood, we have found that patients with higher DD level in the portal vein had longer mean OS than patients with lower ones. The shorter OS has been reported in PDAC as well as in the other cancers with higher DD concentration in the peripheral blood. Fukumoto et al. found that elevated DD concentration in peripheral blood predicts shorter OS in resected non-small cell lung cancer independently of age, gender, stage of the tumor, and level of circulating carcinonembryonic antigen [6]. Ay et al. likewise find elevated DD to predict poor prognosis in all studied solid organ tumors [7]. This clearly shows that D-Dimer levels in the portal and peripheral blood cannot be treated as equivalent.

In pancreatic cancer, not extending the barrier of retroperitoneal space or metastasizing to the liver, all venous blood from the tumor has to be drained to the portal circulation. Therefore, in such cases, portal blood D-dimer concentration reflects fibrinolysis at the site of the primary pancreatic cancer.

As we have previously demonstrated in many cases of pancreatic cancer that did not cross the retroperitoneal barrier and did not metastasize to liver, the D-dimer levels in the portal vein are several times greater than those in the peripheral blood. This cannot be explained only by the phenomenon of dilution of portal blood in the general circulation, because only about 20% of the total volume of circulating blood passes through the portal system.

We hypothesized and confirmed that liver acts as a filter for portal DD by excreting them into bile. The concentration of D-dimers in the peripheral blood is significantly increased when the pancreatic cancer breaks the liver barrier or the tumor begins to infiltrate the retroperitoneal space, hence, the high D-dimer levels in both portal and peripheral blood in patients with metastatic pancreatic tumor. The latter reflect mostly fibrinolysis of extra-gastrointestinal metastases and circulating tumor cells [8].

Directly, very high portal DD concentration may also confirm previously proposed hypothesis of fibrin scaffold surrounding pancreatic cancer from host’s immune system response [9]. On this basis, longer OS time in patients with higher intraoperative portal blood DD concentrations can be explained.

Our study adds to the growing body of evidence that D-dimer concentrations have potential as prognostic biomarkers in pancreatic ductal adenocarcinoma. Further investigation is necessary to both confirm our results in a larger patient population and elucidate the mechanism for the correlation between portal blood D-dimer concentrations and survival time. Along with other authors [10], we conclude that portal circulation is characterized by unique, biological environment that requires further evaluation.

## References

[1] Ballehaninna UK, Chamberlain RS (2012). The clinical utility of serum CA 19-9 in the diagnosis, prognosis and management of pancreatic adenocarcinoma: an evidence based appraisal. J Gastrointest Oncol.

[2] Cao J, Fu Z, Gao L, Wang X, Cheng S, Wang X (2017). Evaluation of serum D-dimer, fibrinogen, and CA19-9 for postoperative monitoring and survival prediction in resectable pancreatic carcinoma. World J Surg Oncol.

[3] Liu P, Zhu Y, Liu L (2015). Elevated pretreatment plasma D-dimer levels and platelet counts predict poor prognosis in pancreatic adenocarcinoma. Onco Targets Ther.

[4] Hong T, Shen D, Chen X, Wu X, Hua D. Preoperative plasma fibrinogen, but not D-dimer might represent a prognostic factor in non-metastatic colorectal cancer: a prospective cohort study. Cancer Biomark. 2017. [Epub ahead of print]. 10.3233/CBM-160510 PMC13020701 28269756

[5] Durczynski A, Kumor A, Hogendorf P, Szymanski D, Grzelak P, Strzelczyk J (2014). Preoperative high level of D-dimers predicts unresectability of pancreatic head cancer. World J Gastroenterol.

[6] Fukumoto A, Taniguchi T, Usami N, Kawaguchi K, Fukui T, Ishiguro F (2015). The preoperative plasma D-dimer level is an independent prognostic factor in patients with completely resected non-small cell lung cancer. Surg Today.

[7] Ay C, Dunkler D, Pirker J, Thaler J, Quehenberger P, Wagner O (2012). High D-dimer levels are associated with poor prognosis in cancer patients. Haematologica.

[8] Durczynski A, Kumor A, Hogendorf P, Szymanski D, Poznanska G, Grzelak P (2014). D-dimers revisited: a new marker of pancreatic cancer. Am J Clin Oncol.

[9] Durczynski A, Kumor A, Grzelak P, Strzelczyk M, Hogendorf P, Poznanska G (2017). Concentration of D-dimers in bile—a novel marker enhancing accuracy of standard CA19-9 measurement: dual test hypothesis. Pancreas.

[10] Arnoletti JP, Zhu X, Almodavar AJO, Veldhuis PP, Sause R, Griffith E (2017). Portal venous blood circulation supports immunosupressive environment and pancreatic cancer circulating tumor cell activation. Pancreas.

